# The Value of the Frog Lateral View Radiograph for Detecting Collapse of Femur Head Necrosis: A Retrospective Study of 1001 Cases

**DOI:** 10.3389/fmed.2022.811644

**Published:** 2022-03-29

**Authors:** Fan Yang, Xiaoqiang Deng, Pengfei Xin, Zhinan Hong, Fengxiang Pang, Wei He, Qiushi Wei, Ziqi Li

**Affiliations:** ^1^Laboratory of Orthopaedics and Traumatology, Lingnan Medical Research Center, Guangzhou University of Chinese Medicine, Guangzhou, China; ^2^Department of Orthopedics, Sun Yat-sen Memorial Hospital, Sun Yat-sen University, Guangzhou, China; ^3^Department of Orthopedics, The Second Affiliated Hospital of Guangzhou University of Chinese Medicine, Guangzhou, China; ^4^Department of Joint Diseases, The Third Affiliated Hospital of Guangzhou University of Chinese Medicine, Guangzhou, China

**Keywords:** femur head necrosis, radiograph, frog lateral view, collapse, diagnosis

## Abstract

**Aims:**

The collapse in femur head necrosis is generally detected by CT or MRI which are not primary routine examination at every follow-up in developing countries. The purpose of this study was to verify the reliability of the frog lateral view radiograph in detecting the collapse of femoral head.

**Methods:**

We retrospectively included 1001 hips of 620 patients with femur head necrosis. The anteroposterior view and frog lateral view of X-ray standard radiographs, CT and MRI of patients were collected and simultaneously evaluated by three orthopedists to evaluate the condition of collapse according to the unified standard. The inter-observer reliability of each view of X-ray for detecting the collapse were analyzed through the weighted Cohen's kappa index. The sensitivity, specificity, positive predictive value, negative predictive value and accuracy of each evaluation method were also calculated.

**Results:**

A moderate or substantial reliability was indicated in the evaluation of frog lateral view radiograph, whereas the anteroposterior view only showed fair or poor reliability. Using the CT or MRI results of collapse as the gold standard, the frog lateral view indicated higher sensitivity and accuracy than the anteroposterior view (sensitivity: 82.8 vs. 64.9%; accuracy: 87.1 vs. 73.9%). The combination of the anteroposterior view and frog lateral view indicated higher reliability than individual views.

**Conclusion:**

The frog lateral view radiograph has higher sensitivity and accuracy than anteroposterior view. It is a complementary method to AP view for detecting the collapse in femur head necrosis during the follow-up, which has moderate or substantial inter-observer reliability.

## Introduction

Femur head necrosis (FHN) is a debilitating disease that could result in lower limb dysfunction (1–3). Due to the high prevalence of FHN in young population, hip arthroplasty is not recommended in most cases because of the high risk of revision surgery (3–6). Therefore, hip-preservation treatments are research hotspots, and early diagnosis and prediction of collapse are equally significant (7). Collapse of the femoral head has been generally viewed as the turning point of disease progression and also affects the treatment options (1, 2). It mainly occurs in the lateral and anterior parts of the femoral head, and could be detected by radiographs, computerized tomography (CT) and magnetic resonance imaging (MRI) with different indications, such as discontinuous femoral head surface, change of head shape and subchondral fracture (8, 9).

Once collapse of the femoral head occurs, patients treated with hip-preservation treatments could have poor prognosis and could require arthroplasty at a very young age (4–6). Therefore, collapse of the femoral head has been the main basis for the disease staging systems, such as Ficat (10), Steinberg (11) and ARCO (12), and also for treatment decisions (13). The above-mentioned staging systems of FHN predominantly detected disease progression *via* anteroposterior (AP) view of standard radiograph, CT or MRI. The AP view of radiograph is more frequently used, but has lower precision than CT and MRI (14). Most importantly, the AP view failed to detect the anterior status of the necrotic lesion, which is also found to be significant for collapse progress through MRI (8). However, MRI is not generally performed at every follow-up due to the high cost and technical requirement. CT is also not recommended for frequent use given the high levels of radiation. In most developing countries like China, CT and MRI examinations do not have distribution equity.

The frog lateral (FL) view of X-ray standard radiographs can be performed in most primary health care systems, whose efficiency in evaluating the anterolateral femoral head-neck junction has been verified in patients with femoroacetabular impingement (15). For the Legg–Calvé–Perthes disease and slipped capital femoral epiphysis in children, the FL view also demonstrated high sensitivity in detection of the early signs and contributed to diagnosis (16). It also allows surgeons to evaluate the anterior femoral head status, which is not possible in AP view. Thus, we hypothesized that the FL view could additionally increase the sensitivity in detection of femoral head collapse. The current study aimed to investigate the reliability of radiographs, including the FL view, the AP view and the combination of them, for detection of the collapse, and whether the combination of AP view and FL view as a routine and primary strategy for detection of femoral head collapse, can achieve better or equivalent reliability compared to CT or MRI.

## Methods

This cross-sectional study was approved by our institutional review board. Because the data was retrospectively collected, the inform consent statement was waived by ethics committee. From December 1, 2016 to August 1, 2020, 1001 hips of 620 patients diagnosed with FHN at our institution were included according to the following inclusion criteria: ① patients with no other complications that would affect the subchondral bone; ② patients who had not received hip-preservation surgery after diagnosis; and ③ patients who underwent the AP view and FL view radiography and either CT or MRI during the same hospitalization.

### Radiographic Data

All included patients underwent standard X-ray radiography, including the AP view and the FL view, and either CT or MRI to detect collapse or subchondral fracture of the involved femoral head. The AP view was performed in erect position. Patients were instructed to stand upright with both feet shoulder width apart and the knees facing forward. The center of the X-ray beam was on the symphysis pubis in the vertical midline, and the field included both hips and iliac crests. For the FL view, patients were positioned supine on the X-ray table and bilateral knees were flexed to let the feet up to the level of knees. Then both legs were abducted and externally rotated while the plane of the pelvis was kept parallel to the plane of the table. The X-ray beam was directed anterior to posterior and centered on the symphysis pubis, while both femoral heads and the great trochanters were included.

CT and MRI were simultaneously performed with the X-ray standard radiographs at our department. MRI examination was performed on a 3-T system (Achieva 3.0T; Philips Medical Systems, Best, The Netherlands). Coronal and oblique axial planes on T1-weighted images and fat-suppressed T2-weighted images were obtained in the supine position with 5 mm slice thickness, 1 mm inter-slice gap and a field of view of 360 × 360 mm. CT examination was performed on *Discovery CT750 HD* (GE Healthcare, Chicago, United States). The images were acquired using the gemstone spectral imaging (GSI) scan mode. Imaging parameters were as follows: tube voltage, 80 kV/140 kV; tube current, 640 mA. Multiplanar reformations of both hips were obtained in the transverse, coronal and sagittal planes, with a section thickness of 1.25 mm.

### Image Evaluation

First, the image signs of hips and pelvis were generally measured by radiologists to exclude changes that potentially affect the femoral head. Then all the included images were analyzed in consensus by three orthopedists, who were randomly selected from the orthopedic department, to measure the collapse extent of femoral head. The inter-observer reliability was analyzed among these three orthopedists by independently evaluating 32 hips, which were also selected randomly from the 1001 hips. Then they were asked to evaluated the 1001 hips together given the difficulty of measurement of large samples. Any disagreement was immediately discussed to achieve a consensus.

For the X-ray standard radiographs, the degree of collapse was evaluated by concentric circles on both AP and FL views using Image J software (1.52a, National Institutes of Health, USA), according to previous studies (16, 17). The crescent sign was also regarded as a sign of collapse. In CT and MRI examinations, the collapse was defined when any slice appeared as discontinuous femoral head surface or subchondral fracture.

### Data Analysis

According to the ARCO 2019 classification of ONFH, the stage 3 is defined as collapsed femoral head. The gold standard to detect the collapsed femoral head was decided by CT or MRI. If either CT or MRI showed a sign of collapse, the case was defined as collapsed femoral head. The statistical analysis was performed using SPSS Statistics 26. The weighted Cohen's kappa index was used to investigate the inter-observer reliabilities between two observers according to the following rules: 1 indicating perfect, 0.81 almost perfect, 0.80–0.61 substantial, 0.60–0.41 moderate, 0.40–0.21 fair, and <0.20 poor. The sample size used to calculate the kappa value was estimated by PASS 2021 version based on the preliminary experiment. The sensitivity, specificity, positive predictive value (PPV), negative predictive value (NPV) and accuracy of AP view, and FL view were calculated, respectively. The statistical difference between them was evaluated by McNemar test.

## Results

### The Basic Data of Included Cases

The original data is available through contracting with the corresponding author. The 1001 hips from 620 patients, with the mean age of 36.95 years (17–85 years). Of these, 317 patients (519 hips) had a history of steroid administration, 219 patients (353 hips) had a history of alcohol abuse, 15 patients (17 hips) had a history of femoral neck fracture, and the remaining 69 patients (112 hips) had no relevant history (idiopathic). Eighteen included hips from 12 patients had no CT results, and 293 hips from 185 patients had no MRI results. Based on CT or MRI examination, 308 hips were classified as ARCO stage II, 460 hips as stage IIIA, and 233 hips as stage IIIB.

### Inter-reliability of Radiograph for Detection of Collapse

The inter-reliability of three orthopedists for evaluating 32 hips randomly selected from the total 1001 hips indicated a moderate or substantial reliability in evaluating the FL view, but only fair or poor reliability in evaluating the AP view (Table 1). For the reasons contribute to the inconsistency, there were 14 hips that have disagreements as to whether they are ARCO stage 2 (pre-collapse) or stage 3 (collapsed), which was the most frequent inconsistency. And 12 hips of them appeared inconsistency in AP view, but only 6 hips in FL view, indicating the FL view is more obvious for detecting the collapse.

**Table 1:** The Kappa value among observers for evaluating the AP view and FL view.

	**AP view**	**FL view**
Observer 1 vs. 2	0.122	0.565
Observer 1 vs. 3	0.345	0.552
Observer 2 vs. 3	0.304	0.639

### The Reliability of AP View and FL View for Detection of Collapse

**Figure 1:**
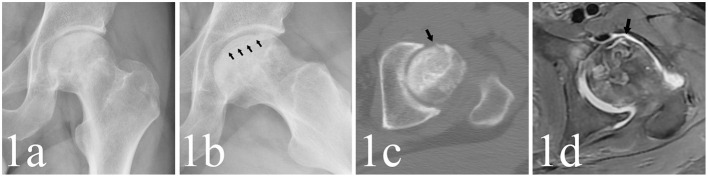
**(a,b)** A 29-year-old male patient whose left hip appeared as crescent sign and collapsed in FL view (as the black arrows point) but not in AP view. **(c,d)** The CT and MRI detected subchondral fracture in anterior femoral head (as the black arrows point).

**Figure 2:**
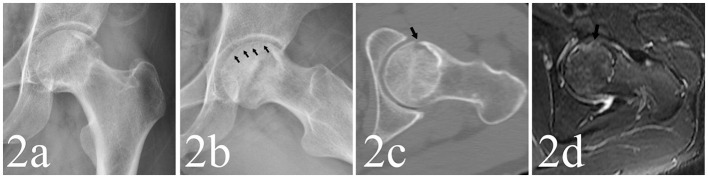
**(a,b)** A 33-year-old male patient whose left hip appeared as crescent sign in FL view (as the black arrows point) but not in AP view. **(c,d)** The CT and MRI also detected subchondral fracture in anterior part of femoral head (as the black arrows point).

**Figure 3:**
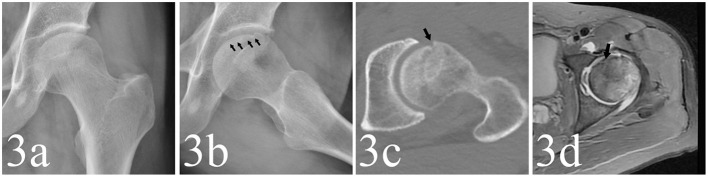
**(a,b)** A 48-year-old male patient whose left hip appeared to be collapsed in FL view (as the black arrows point) but not in AP view. **(c,d)** The CT and MRI detected the collapse in anterior femoral head (as the black arrows point).

For the detection of collapse, the FL view radiograph showed collapse in 617 hips, of which 168 hips showed no collapse in AP view. The AP view indicated collapse in 483 hips, of which 34 showed no collapse in FL view. The radiographs and MRI are shown in Figures 1–3. Compared with the gold standard, the sensitivity, specificity, PPV, NPV and accuracy of AP view were 64.9, 100, 100, 49.6, 73.9%, respectively, while the FL view were 82.8, 99.6, 99.8, 66.7, 87.1%, respectively. The McNemar test indicated that the sensitivity of FL view was significantly higher than AP view (*p* < 0.001). There was no statistical difference in specificity between them (Table 2).

**Table 2:** Comparation between standard radiograph and golden standard.

		**Collapse based** **on CT or MR**		**Total**
		**Negative**	**Positive**	
Collapse based on AP view	Negative	257	261	518
	Positive	0	483	483
Collapse based on FL view	Negative	256	128	384
	Positive	1	616	617
Collapse based on AP and FL view	Negative	256	94	350
	Positive	1	650	651
Total		257	744	1,001^a^

a *The total number of the included cases*.

By combining the AP view and the FL view to diagnose the collapse, the sensitivity, specificity, PPV, NPV and accuracy were 87, 100, 100, 73, 90%, respectively. The sensitivity was also higher than AP view or FL view alone (*p* < 0.001).

### The Reliability of AP View and FL View for Detection of Crescent Sign

A total of 149 cases showed crescent sign positive in FL view but negative in AP view (Table 3). However, 13 negative cases in FL view were detected positive in AP view, indicating that the crescent sign was more frequently detected in FL view (Figures 1–3).

**Table 3:** Comparation of crescent sign between AP and FL view.

		**Crescent sign on FL view**		**Total**
		**Negative**	**Positive**	
Crescent sign on AP view	Negative	804	149	953
	Positive	13	35	48
Total		817	184	1,001

In the 804 cases without crescent sign in both AP and FL views (Table 4), the sensitivity, specificity, PPV, NPV and accuracy of AP view were 64.2, 100, 100, 56.7, and 75.6%, and that of FL view were 77.3, 99.6, 99.8, 67.4, and 84.5%, respectively. The sensitivity and accuracy of FL view remained significantly higher than AP view (*p* < 0.001), indicating that the FL view had higher sensitivity in detecting the other collapse signs besides the crescent sign, such as discontinuous femoral head surface or the deformation of femoral head.

**Table 4:** Comparation between standard radiograph without crescent sign and golden standard.

		**Collapse based on CT or MR**		**Total**
		**Negative**	**Positive**	
Collapse based on AP view	Negative	257	196	453
	Positive	0	351	351
Collapse based on FL view	Negative	256	124	380
	Positive	1	423	424
Total		257	547	804^a^

a *The total number of the cases without crescent sign in standard radiograph*.

## Discussion

The detection of collapse in FHN is critical for the prognosis and treatment strategy. The status of cartilage and structure inside intact femoral head are considered to be better than collapsed femoral head (17). Many studies have attempted to find a precise and practical way to detect or predict the collapse in the process of FHN, and have suggested several methods that could accurately evaluate the risk of collapse (7, 18, 19). Most of them depend on the measurement of necrotic volume or the necrosis location, which are usually evaluated through MRI (14, 20, 21).

In recent years, anterior necrosis has received extensive attention worldwide as MRI is more frequently used in clinical practice (8). However, due to the unavailability of MRI in most areas in China and other developing countries, patients typically need to wait for a long time to perform an MRI in cities far away from their homes (22). Furthermore, patients need to follow-up every 3 months to determine the progression of FHN (23), which increases the financial burden for the majority of patients who have no health insurance. CT, another precise way to detect collapse, is not recommended to be performed frequently due to radiation exposure. Hence, it is critical to find a method to better detect the collapse in anterior femoral head.

The FL view of X-ray standard radiographs has been recently reported as another way to visualize the anterior necrosis and predict the prognosis (24). This method has been clinically used in our department for more than 10 years. The present study first analyzed the effectiveness of FL view to detect collapse of anterior necrosis through large sample size, and also evaluated the inter-reliability of this method. The results showed that the inter-reliability of FL view was significantly higher than AP view, with higher sensitivity and accuracy than AP view, when CT and MRI were considered as the gold standard. Combination of the AP and FL views to detect collapse showed higher sensitivity and accuracy than individual views. Therefore, as a practical and economical method, the FL view can improve the accuracy of detection of collapse throughout the follow-up of FHN.

There are several potential advantages of FL view for detecting collapse. First, the necrosis area usually accounts for more volume in the anterior femoral head, which could be obviously indicated by the FL view (8, 16). Through 3D analysis of MRI, the anterior area also appeared vulnerable to necrosis (8). This is related to an alteration of the vascularization of the fine blood vessels that irrigate the anterior and superior parts of the femoral head (25). Furthermore, the realistic load analysis of hip indicated that the femoral head receives higher peak force when walking and climbing stairs, which often puts the anterior femoral head under the interface stresses (26). Due to the larger necrotic volume and higher interface stress, the anterior femoral head appears to be at a higher risk of collapse than other parts. We evaluated the crescent sign in both AP and FL views, and the FL view was more sensitive for detecting the crescent sign than AP view, indicating a higher sensitivity in finding the collapse in early stages. This should contribute to the clinical decision for dealing with the collapse.

In clinical practice, after the first diagnosis of FHN through plain radiography, CT and MRI, the FL view has been used as a complementary method during the follow-up to observe the anterior femoral head in our department for more than 10 years (27). It can be easily performed even in a basic hospital, which is easily accessed by patients than a large central hospital. This facilitates the follow-up process every 3 months, and has been efficiently conducted in the past studies on FHN (27). The inter-observer reliability, another value for practical use, showed better results in FL view than AP view. This was partly due to the higher frequency of collapse in the anterior part of femoral head, which could be clearly indicated in FL view but was indistinct in AP view. By combining the two views together, the collapse sign could be precisely detected during follow-up until the next CT or MRI was performed, which should be performed every 6 months (23).

There were two main limitations of this study. First, given the large sample size and the difficulty to evaluate 1001 hips, the measurement was performed by the consensus of three orthopedists together, but not independently. This may have caused potential evaluation bias though the inter-observer reliability was tested. Second, some patients only had CT or MRI data, which may have led to missed detection of collapse.

## Conclusion

The FL view is an effective and practical complementary method to AP view for detecting the collapse in FHN during the follow-up. It can precisely detect the anterior collapse of femoral head, which may be indistinct in AP view. Combination of AP and FL views should be recommended as routine examination during the primary diagnosis and routine follow-up of FHN patients.

## Summary

### Article Focus

The detection of femoral head collapse is significant in therapeutic strategy for femur head necrosis. CT and MRI could precisely detect the collapse but lack popularity at every follow-up in developing countries. The study focused on the diagnostic reliability and accuracy of frog lateral view radiograph, a primary routine examination, for detecting the collapse in femur head necrosis. We hypothesized that the frog lateral view radiograph could improve the reliability and accuracy of radiograph (anteroposterior view) and get similar accuracy with CT or MRI.

### Key Message

Due to the limited distribution equity of CT and MRI in developing country, a new standard protocol for detection of collapse should be studied. Frog lateral view radiograph have higher reliability and accuracy than anteroposterior view. The study indicated the combination of frog lateral view and anteroposterior view radiograph has adequate accuracy in detecting the collapse at every follow-up compared with CT or MRI.

### Strengths and Limitations of This Study

The study included the largest sample size in previous studies. The evaluation progress was standardized and the inter-observer reliability was measured. However, some patients only had one of CT and MRI data, which may have led to missed detection of collapse and cause potential risk of bias.

## Data Availability Statement

The original contributions presented in the study are included in the article/Supplementary Materials, further inquiries can be directed to the corresponding author.

## Ethics Statement

The studies involving human participants were reviewed and approved by the Ethics Committee of the Third Affiliated Hospital of Guanghzou University of Chinese Medicine. The patients/participants provided their written informed consent to participate in this study.

## Author Contributions

FY, WH, and ZL designed the clinical study. FY, XD, PX, ZH, FP, WH, QW, and ZL contributed to the clinical work. FY and ZL conducted the data collection, data analysis, and wrote the paper. WH and ZL gained the funding. All authors have read and approved the final submitted manuscript.

## Funding

This work was supported by National Natural Science Foundation of China (Grant Numbers 81904226 and 81873327).

## Conflict of Interest

The authors declare that the research was conducted in the absence of any commercial or financial relationships that could be construed as a potential conflict of interest.

## Publisher's Note

All claims expressed in this article are solely those of the authors and do not necessarily represent those of their affiliated organizations, or those of the publisher, the editors and the reviewers. Any product that may be evaluated in this article, or claim that may be made by its manufacturer, is not guaranteed or endorsed by the publisher.

## Supplementary Material

Table S1 The original data of measurement.
